# An explorative epigenome-wide association study of plasma renin and aldosterone concentration in a Ghanaian population: the RODAM study

**DOI:** 10.1186/s13148-022-01378-5

**Published:** 2022-12-01

**Authors:** Eva L. van der Linden, Adrienne Halley, Karlijn A. C. Meeks, Felix Chilunga, Charles Hayfron-Benjamin, Andrea Venema, Ingrid M. Garrelds, A. H. Jan Danser, Bert-Jan van den Born, Peter Henneman, Charles Agyemang

**Affiliations:** 1 Department of Public and Occupational Health, Amsterdam UMC, University of Amsterdam, Amsterdam Public Health Research Institute, Location AMC, Meibergdreef 9, 1105AZ Amsterdam, The Netherlands; 2 Department of Vascular Medicine, Amsterdam UMC, University of Amsterdam, Amsterdam Cardiovascular Sciences, Amsterdam, The Netherlands; 3 Center for Research on Genomics and Global Health, National Human Genome Research Institute, National Institutes of Health, Bethesda, MD USA; 4 Department of Physiology, University of Ghana Medical School, Accra, Ghana; 5 Department of Anesthesia and Critical Care, Korle Bu Teaching Hospital, Accra, Ghana; 6 Department of Human Genetics, Genome Diagnostics Laboratory Amsterdam, Amsterdam UMC, University of Amsterdam, Amsterdam Reproduction and Development, Amsterdam, The Netherlands; 7 Division of Pharmacology and Vascular Medicine, Department of Internal Medicine, Erasmus MC, University Medical Center Rotterdam, Amsterdam, The Netherlands

**Keywords:** DNA methylation, Epigenome-wide association study, Renin, Aldosterone, RAAS, Hypertension, Sub-Saharan Africa, RODAM

## Abstract

**Background:**

The epigenetic regulation of the renin–angiotensin–aldosterone system (RAAS) potentially plays a role in the pathophysiology underlying the high burden of hypertension in sub-Saharan Africans (SSA). Here we report the first epigenome-wide association study (EWAS) of plasma renin and aldosterone concentrations and the aldosterone-to-renin ratio (ARR).

**Methods:**

Epigenome-wide DNA methylation was measured using the Illumina 450K array on whole blood samples of 68 Ghanaians. Differentially methylated positions (DMPs) were assessed for plasma renin concentration, aldosterone, and ARR using linear regression models adjusted for age, sex, body mass index, diabetes mellitus, hypertension, and technical covariates. Additionally, we extracted methylation loci previously associated with hypertension, kidney function, or that were annotated to RAAS-related genes and associated these with renin and aldosterone concentration.

**Results:**

We identified one DMP for renin, ten DMPs for aldosterone, and one DMP associated with ARR. Top DMPs were annotated to the *PTPRN2, SKIL,* and *KCNT1* genes, which have been reported in relation to cardiometabolic risk factors, atherosclerosis, and sodium-potassium handling. Moreover, EWAS loci previously associated with hypertension, kidney function, or RAAS-related genes were also associated with renin, aldosterone, and ARR.

**Conclusion:**

In this first EWAS on RAAS hormones, we identified DMPs associated with renin, aldosterone, and ARR in a SSA population. These findings are a first step in understanding the role of DNA methylation in regulation of the RAAS in general and in a SSA population specifically. Replication and translational studies are needed to establish the role of these DMPs in the hypertension burden in SSA populations.

**Supplementary Information:**

The online version contains supplementary material available at 10.1186/s13148-022-01378-5.

## Background

Sub-Saharan African (SSA) origin populations face a higher burden of hypertension compared with other ethnic groups [1], and African migrants in Europe have a much higher prevalence of hypertension than their non-migrating counterparts in Africa [2]. Salt sensitivity (SS) is one of the best studied pathophysiological mechanisms to explain the high prevalence of hypertension among SSA origin populations [3]. SS refers to an increase in blood pressure upon salt intake and relates to excessive sodium and water retention by the kidneys, and suppression of the renin-angiotensin-aldosterone system (RAAS) [4], a physiological system regulating circulating blood volume and blood pressure [5]. West African descent populations have higher prevalence of salt-sensitive hypertension [6], with a profile of suppressed renin, accompanied by an increased aldosterone-to-renin ratio [7]. SS is affected by multiple factors including genetics [8], and phenotypical aspects such as age, obesity, insulin resistance, and hypertension [9]. However, key underlying determinants and exact molecular mechanisms of SS among SSA populations still need to be elucidated. In this light, studying epigenetic modifications in relation to RAAS can be of interest. ‘Epigenetics’ refers to changes in readability and transcription of the genome, without alterations to the DNA sequence itself [10]. Epigenetic modifications, of which DNA methylation is the most widely studied epigenetic feature, are influenced by both genetic and environmental factors. Several studies have identified differentially methylated positions (DMPs) located in RAAS-related genes to be associated with hypertension [11, 12], including the *AGT*, *AGTR1*, *ACE, NOS3,* and *SCNN1A* genes [5, 13]. However, none of these studies have examined the association between epigenome-wide DNA methylation and concentration of RAAS hormones. Moreover, studies that identified RAAS-related genes were mainly conducted in European and African American populations, whereas these groups differ from SSA based populations in terms of genetics and environment. Therefore, the aim of this proof-of-principle study was to assess the association between DNA methylation and plasma renin and aldosterone concentrations, and aldosterone-to-renin ratio (ARR) in Ghanaians.

## Methods

### Study population and study design

For this study, baseline data of the longitudinal, multicentre Research on Obesity and Diabetes among African Migrants (RODAM) study were used. Details on the RODAM study have been published before [14] and will be briefly described here.

In the period 2012–2015, 6385 Ghanaian men and women were recruited. The participants resided in rural Ghana, urban Ghana, and in the European cities of London, Amsterdam, and Berlin. The majority of participants were of Akan ethnicity, and Ghanaians in Europe were first-generation migrants.

Of participants aged 25 years and older with complete data on physical examination and blood samples profile, 736 participants were selected for DNA methylation profiling. These participants were selected based on a case-control design, including about 300 cases with non-drug-treated diabetes, 300 controls without diabetes, and 135 controls with neither diabetes nor obesity. After excluding participants with sex discordances (*n* = 11), duplicates (*n* = 8), and those not meeting the quality control thresholds (*n* = 12), 713 eligible participants remained. Of these participants, those without aldosterone and renin measurements were excluded (*n* = 644, excluding all participants residing in London and Berlin as they were not included in aldosterone and renin analysis), as was one participant with an outlier in renin concentration (77 pg/mL), which was > 3 times the SD of the log-transformed renin concentration. This resulted in 68 participants in the current analysis, all residing in rural and urban Ghana, or Amsterdam (Additional file 1: FigureS1).

### Phenotypic measurements

Data collection procedures for questionnaire and physical examination were highly standardised across the different study locations. Questionnaires were used to collect data on gender, age, level of education, and length of stay in Europe. Physical examination was performed using validated devices. Weight was measured in light clothing without shoes with a SECA 877 scale to the nearest 0.1 kg. Height was measured without shoes using a SECA 2017 portable stadiometer to the nearest 0.1 cm. Anthropometric measures were taken twice and the mean of the two measures was used in analyses. Body mass index (BMI) was calculated by dividing the weight in kilograms by the height in metres squared. After at least 5 min of rest, three blood pressure (BP) readings were taken in a sitting position, with a cuff fixed on the left arm. The mean of the second and third reading was used in the analyses. Hypertension was defined as having a BP of systolic ≥ 140 mmHg and/or diastolic ≥ 90 mmHg and/or the use of BP-lowering medication. BP-lowering medication was categorised based on Anatomical Therapeutic Chemical classification of medication that participants brought with them to the research location.

Venous blood samples were collected in the sitting position, after an overnight fast of at least ten hours. All samples were collected around the same time of the day (morning) to control for the influence of circadian rhythm. After samples collection, samples were immediately processed, aliquoted and cryopreserved, before storage in − 80˚C freezers. Samples collected in Ghana were shipped to Europe while kept frozen at − 80 ˚C. Fasting plasma glucose concentrations were measured using the hexokinase method by colorimetry, in the laboratory of the Institute of Tropical Medicine and International Health, Berlin, Germany. Diabetes mellitus was defined according to self-reported diabetes and/or fasting glucose ≥ 7.0 mmol/L. Aldosterone and renin concentration (pg/mL) were measured in heparin plasma samples, and analyses were performed at the Department of Internal Medicine of the Erasmus MC, Rotterdam, the Netherlands. All samples, including those from Ghana, were analysed in the same laboratory, to prevent inter-laboratory differences affecting the results. After thawing of the samples, renin concentration was determined by an immunoradiometric assay (Cisbio, Saclay, France) using an active site-directed radiolabelled antibody binding to renin only. The lower detection limit of this assay was 2 pg/mL. Aldosterone concentrations were measured by solid-phase radioimmunoassay (Demeditec Diagnostics, Kiel, Germany), with a lower detection limit of 12 pg/ml. Aldosterone-to-renin ratio (ARR, pg/mL/pg/mL) was calculated by dividing aldosterone by the renin concentration. The distributions of aldosterone, renin, and ARR were assessed using histograms and the Shapiro-Wilkinson test. To ensure normal distribution of the traits, aldosterone concentration was transformed using Box-Cox transformation, and renin and ARR were log-transformed. Renin, aldosterone and ARR were chosen for analysis, because of their relevance in the context of SS and salt-sensitive hypertension.

### DNA methylation profiling, processing, and quality control

Previous RODAM publications have elaborated upon the DNA methylation profiling, processing, and quality control on whole blood samples [15], and this process will be summarised here. In the lab of Source BioScience, Nottingham, UK, the Zymo EZ DNAm™ kit was used for bisulfite conversion of DNA. Using the Infinium® HumanMethylation450 BeadChip, the converted DNA was amplified and hybridised, hereby quantifying DNAm levels of approximately 485,000 CpG sites. Methylation levels were measured based on the intensities of the methylated and unmethylated probes for each CpG site on the array. These intensities were expressed as methylation Beta values, which is a value between zero (unmethylated) and one (methylated). A log2 ratio of the intensities of methylated versus unmethylated probes was calculated, which is referred to as *M* values. Using R statistical software (version 4.2.0), quality control was performed using the *MethylAid* package (version 1.30.0), using default thresholds of 10.5 for methylated and unmethylated intensities, 11.75 for overall quality control, 12.75 for bisulfite conversion, 12.50 for hybridisation control, and 0.95 for detection *p*-value. Functional normalisation of the raw 450 K data was conducted using the *minfi* package (version 1.42.0). After the removal of probes annotated to the X and Y chromosomes, known to involve cross-hybridisation or to contain common single-nucleotide polymorphisms (SNPs) with a minor allele frequency of ≥ 5%, a set of 429,449 CpG sites remained for analysis [16]. Blood cell type distribution was estimated based on the method of Houseman et al. [17].

### Statistical analyses

#### Association between renin, aldosterone, and ARR and DNA methylation

To assess differentially methylated positions (DMPs), multivariate linear regression was performed between renin concentration, aldosterone, and ARR (independent variables) and DNA methylation *M* values (dependent variable), using the *Limma* package (version 3.52.0). Methylation *M* values were used in statistical analysis to ensure normal distribution, whereas methylation Beta values were used to facilitate interpretation and visualisation. Models were adjusted for sex, age, BMI, diabetes mellitus, hypertension, estimated blood cell type counts (CD8 + T lymphocytes, CD4 + T lymphocytes, natural killer cells, B cells, monocytes, granulocytes), and technical covariates (hybridisation batch and array position), because of correlation with DNA methylation in the principal components analysis, as well as because of an overrepresentation of participants with diabetes and high BMI in the sample. Model fit was assessed using QQ plots, as well as the genomic inflation factor lambda. Because of improvement in model fit after bias and inflation correction using the R package *bacon* (version 1.24.0) [18], we applied this adjustment to all analyses (lambda with inflation correction for renin 1.046, aldosterone 0.995, ARR 1.026) (Additional file 1: FigureS2). False discovery rate (FDR) adjustment of the *p*-values was applied, to reduce the risk of false positive findings in multiple testing. An FDR-adjusted *p*-value of < 0.05 was considered epigenome-wide significant.

To identify the contribution of the top DMPs to the variance in renin and aldosterone concentration, linear regression was performed using z-standardised methylation *M* values of the identified DMPs as independent variable and the (untransformed) trait of interest as dependent variable. The *R* squared statistics from the linear regression analyses with and without covariates were used to calculate trait variance explained by each DMP. Similar methods were used to assess the explained variance in systolic and diastolic BP, with the z-standardised *M* values of the top DMPs associated with renin, aldosterone, and ARR as independent variables in the linear regression model, adjusted for age, sex, BMI, diabetes mellitus, blood cell distribution, and technical variates.

As several types of BP-lowering medication can interfere with the RAAS system, a sensitivity analysis excluding those on BP-lowering medication (*n* = 10) was performed, to assess the impact of medication use on the association.

For the top DMPs per trait, we extracted the median methylation Beta values with accompanying interquartile ranges, and stratified these per geographical location, to examine whether differences in methylation levels existed between participants residing in rural and urban Ghana, and Amsterdam. These median values were compared between the location using nonparametric the Kruskal–Wallis test. A two-sided *p*-value < 0.05 was considered statistically significant.

#### Differentially methylated regions

To assess whether DNA methylation of genomic regions, rather than on single positions, was associated with the traits of interest, the *R* package *bumphunter* (version 1.38.0) was used to assess differentially methylated regions (DMRs), using similar models as used in the DMP analysis. Methylation *M* value cut-offs of 0.2 was chosen for the input, which limited the analysis to 100 candidate regions and 20% difference in regression coefficient beta between candidate probes. The analysis was run with bootstrapping with 500 permutations. DMRs with more than two CpGs, and a family-wise error rate (FWER) < 0.2 were considered statistically significant. DMRs were visualised using *coMET* package (version 1.27.2).

#### Replication

We used the EWAS atlas [19] to extract all CpGs previously reported to be associated with BP, systolic blood pressure (SBP), diastolic blood pressure (DBP), and hypertension and performed a candidate DMP analysis. Additionally, we tried to replicate CpG sites significantly associated with eGFR in a large, multi-ethnic, meta-analysis of EWAS [20]. Lastly, we extracted the probes previously annotated to RAAS-related genes (*ACE, AGT, REN, CYP11B2, HDS11B2,* and *NR3C2*) [21] and performed a candidate-gene DMP analysis on these for each of our traits of interest. For these candidate-gene analyses, the same models, including adjustment for bias and inflation, were used as for the main analysis. All CpGs with an FDR-adjusted *p*-value of < 0.05 were considered to be statistically significant.

#### Biological relevance

Gene expression levels for the epigenome-wide significant DMPs were assessed using the publicly available iMethyl database, in which the DNA methylation levels of CD4 + T lymphocytes as well as gene expression levels, denoted per kilo base of transcript per million mapped fragments (FPKM), are reported for 100 apparently healthy individuals living in Japan [22].

Enrichment analysis using the Kyoto Encyclopedia of Genes and Genomes (KEGG) catalogue in package R *missMethyl* (version 1.30.0) was performed to gain insight into the function and biological pathways of our findings. The top 5000 CpGs with the smallest *p*-values per trait were used as input.

The *gaphunter* gfunction of the *minfi* package was used to examine whether the significant DMPs were potentially under the influence of genetic variation. The function was run with a threshold of 0.05, reflecting a gap of 5% in beta value, suggestive of genetic influence.

## Results

### Population characteristics

Table 1 shows the population characteristics. Males and females were included at nearly equal proportions, and the mean age was 52 years. The majority of the participants resided in rural and urban Ghana, and all Ghanaian migrants resided in Amsterdam. One-third of the participants had diabetes mellitus and close to 40% of the participants were classified as hypertensive. Ten participants reported taking prescribed BP-lowering medication, of which calcium channel blockers (*n* = 9) and angiotensin receptor blockers (*n* = 4) were the most used types of drugs. The median renin concentration was 7.62 pg/mL, the median aldosterone was 103.44 pg/mL, and the median ARR was 14.16 pg/mL/pg/mL.

**Table 1:** Population characteristics

*N*	68
Sex, male (%)	32 (47.1)
Age, years (mean (SD))	52.6 (9.0)
Site (%)	
Rural Ghana	24 (35.3)
Urban Ghana	32 (47.1)
Amsterdam	12 (17.6)
BMI, kg/m^2^ (mean (SD))	25.1 (5.5)
Diabetes mellitus (%)	22 (32.4)
Hypertension (%)	27 (39.7)
Systolic blood pressure, mmHg (mean (SD))	131.78 (23.98)
Diastolic blood pressure, mmHg (mean (SD))	82.28 (15.53)
Prescribed BP-lowering medication (%)	10 (14.7)
Diuretics (%)	2 (2.9)
Beta blockers (%)	2 (2.9)
Calcium channel blockers (%)	9 (13.2)
Angiotensin receptor blocker (%)	4 (5.9)
Renin, pg/mL (median [IQR])	7.62 [4.26, 11.62]
Aldosterone, pg/mL (median [IQR])	103.44 [70.85, 163.21]
ARR (pg/mL/pg/mL) (median [IQR])	14.16 [7.45, 28.05]

*SD *Standard deviation, *BMI* Body mass index, *BP* Blood pressure, *ARR* Aldosterone-to-renin ratio, *IQR* Interquartile range

### Differentially methylated positions

#### Renin

**Fig. 1:**
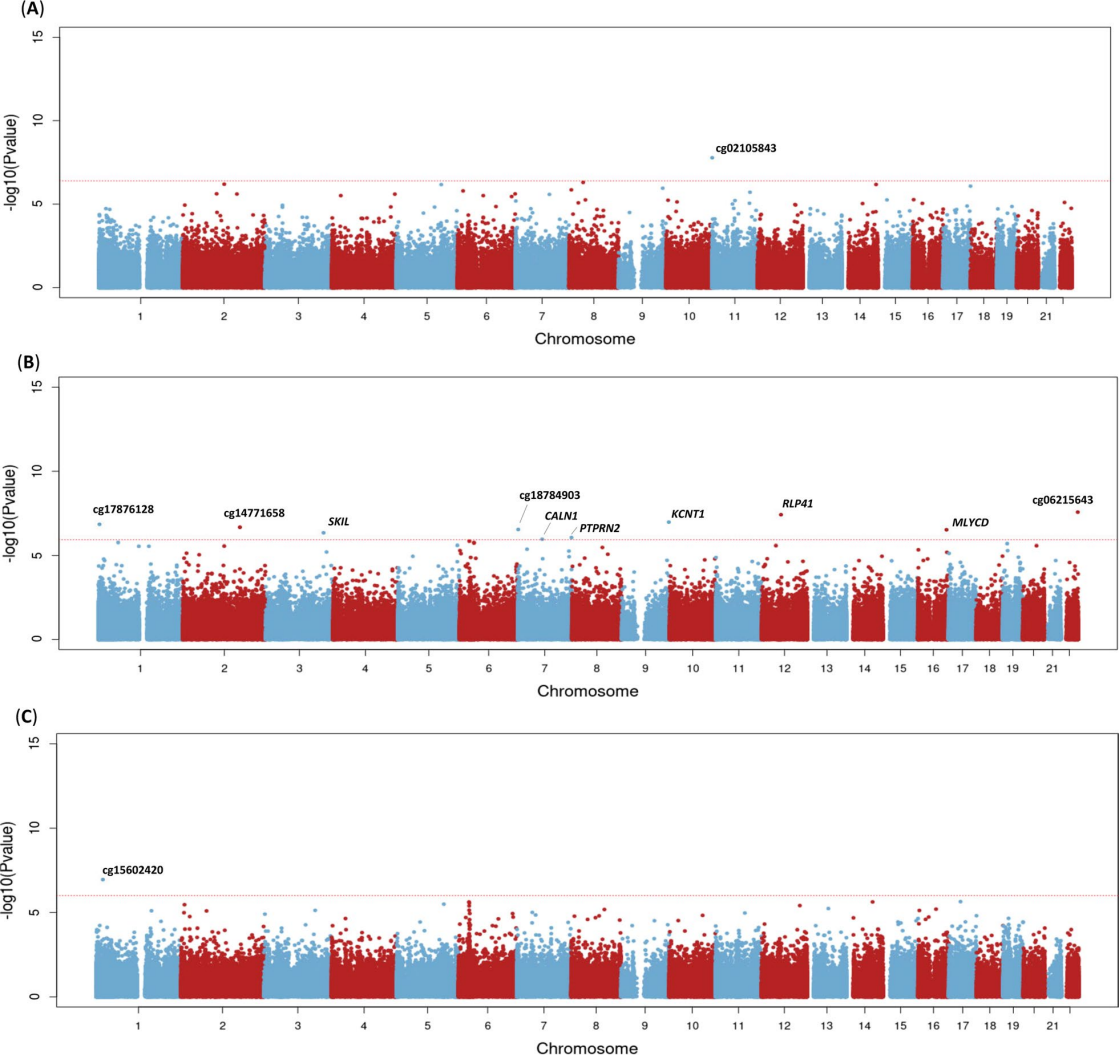
Manhattan plots for bacon adjusted *p*-values of the EWAS of renin **A** aldosterone **B** and aldosterone-to-renin ratio **C.** Dotted lines indicates epigenome-wide significant *p*-value

We found one intergenic CpG, cg02105843, epigenome-wide significantly associated with renin concentration (Table 2, Fig. 1A). One standard deviation (SD) increase in methylation *M*-value of this DMP was associated with 9.08 pg/mL lower renin levels and this DMP contributed to 15% of the variance in renin concentration (Table 3). The top 5 DMPs associated with renin concentration explained 6.4% in variance in SBP and 8.0% in variance in DBP. Excluding participants on BP-lowering medication did not impact this result, and there was no difference in methylation levels for this DMP between participants residing in rural and urban Ghana or in Amsterdam.

**Table 2:** Epigenome-wide significant differentially methylated positions associated with renin, aldosterone and aldosterone/renin ratio

	Regres.Coeff*	*p*-value	FDR adj. *p*-val	chr	pos	Gene symbol**	Gene group	Methylation, % (sd)***
*Renin*								
cg02105843	− 0.149	1.65E-08	0.0071	chr11	522,809	intergenic	90.46 (2.06)	
*Aldosterone*								
cg06215643	0.209	2.73E-08	0.0084	chr22	50,119,046	intergenic	57.05 (5.13)	
cg03123773	− 0.613	3.90E-08	0.0084	chr12	56,510,201	*RPL41*	TSS200	5.83 (3.24)
cg21178689	0.291	1.08E-07	0.0154	chr9	138,594,307	*KCNT1*	Body	64.4 (7.16)
cg17876128	− 0.247	1.46E-07	0.0156	chr1	4,068,541	intergenic	86.45 (4.01)	
cg14771658	0.196	2.16E-07	0.0185	chr2	167,350,883	intergenic	37.57 (5.31)	
cg18784903	0.270	2.90E-07	0.0185	chr7	1,337,353	intergenic	65.02 (6.38)	
cg09869144	0.150	3.01E-07	0.0185	chr16	83,948,818	*MLYCD*	Body	98.18 (0.36)
cg15627834	− 0.489	4.55E-07	0.0245	chr3	170,078,469	*SKIL*	Body	91.89 (9.01)
cg06124066	− 0.148	8.83E-07	0.0421	chr7	157,885,905	*PTPRN2*	Body	91.01 (1.78)
cg24500222	− 0.173	1.10E-06	0.0472	chr7	71,747,028	*CALN1*	5'UTR	76.47 (4.74)
*ARR*								
cg15602420	− 0.217	1.12E-07	0.0481	chr1	20,205,059	Intergenic	77.9 (5.53)	

^*^For *M*-values, model adjusted for covariates: sex, age, BMI, diabetes, hypertension, CD8T, CD4T, NK, Bcell, Monocytes, Granulocytes, batch, position; *ARR*, aldosterone-to-renin-ratio

^**^Annotated using UCSC genome browser, genome build hg37

^***^Methylation level calculated as: methylation beta*100

**Table 3:** Association between z-transformed DNA methylation levels of top differentially methylated positions (independent variable) and renin, aldosterone, and aldosterone-to-renin-ratio concentration (dependent variable)

	chr	pos	Gene symbol	Gene group	Regression coeff	Explained variance (%)
*Renin*						
cg02105843	chr11	522,809	Intergenic		− 9.08	15.24
*Aldosterone*						
cg06215643	chr22	50,119,046			44.16	18.45
cg03123773	chr12	56,510,201	*RPL41*	TSS200	− 59.72	20.43
cg21178689	chr9	138,594,307	*KCNT1*	Body	45.22	17.54
cg17876128	chr1	4,068,541	Intergenic		− 53.90	19.69
cg14771658	chr2	167,350,883	Intergenic		50.93	20.03
cg18784903	chr7	1,337,353	Intergenic		45.30	20.12
cg09869144	chr16	83,948,818	*MLYCD*	Body	73.58	23.4
cg15627834	chr3	170,078,469	*SKIL*	Body	− 56.33	23.15
cg06124066	chr7	157,885,905	*PTPRN2*	Body	− 63.38	17.62
cg24500222	chr7	71,747,028	*CALN1*	5'UTR	− 60.77	16.42
*ARR*						
cg15602420	chr1	20,205,059	intergenic		− 13.25	19.49

Model: trait (untransformed) ~ DNAm(Mval) + sex + age + BMI + diabetes + hypertension + CD8T + CD4T + NK + Bcell + Mono + Gran + batch + position; *ARR*, aldosterone-to-renin-ratio

#### Aldosterone

Ten CpGs were associated with aldosterone concentration on an epigenome-wide level (Table 2, Fig. 1B). The DMPs with the smallest *p*-values were located in an intergenic region (cg06215643), in the transcription start site (TSS) of the *RPL41* gene (cg03123773), and in the body of the *KCNT1* gene. DMPs with the highest explained variance in aldosterone concentration were cg09869144 and cg15627834, located in the body of the *MLYCD* and *SKIL* genes, respectively, contributing to 73.58 and − 56.33 pg/mL change in aldosterone level with one SD increase in *M* value. The combined ten DMPs explained 46% of the variance in aldosterone concentration (Table 3), 19.6% in SBP, and 24.2% in DBP levels.

In the sensitivity analysis excluding ten participants with prescribed BP-lowering medication, four of the ten CpGs did no longer reach epigenome-wide significance. However, these four CpGs, cg17876128, cg14771658, cg18784903, and cg24500222, did show a similar direction of effect and effect size as in the main analysis including participants with BP-lowering medication. For the top DMPs, there was no significant difference in methylation levels between the geographical locations.

##### ARR

ARR was epigenome-wide significantly associated with methylation of intergenic CpG cg15602420 (Table 2, Fig. 1C). An increase of one SD in methylation level of this DMP was associated with 13.25 pg/mL/pg/mL lower ARR and explained 19% of the variance in this trait (Table 3). 6.2% of the variance in SBP and DBP were explained by the top 5 DMPs associated with ARR. Excluding participants with BP-lowering medication did not affect our findings. No difference in methylation level between the different locations of residence could be observed.

### Differentially methylated regions

For renin, we identified two DMRs at an FWER < 0.2, annotated to the *NFYA* (Additional file 1: Figure S3) and the *RNF39* gene (Additional file 1: Figure S4), both located on chromosome 6. One DMR, annotated to the *HDL-DRB1* gene, was associated with aldosterone concentration (Additional file 1: Figure S5). No DMR was identified for the ARR. There was no overlap between the significant DMPs and DMRs.

### Replication

Through the EWAS atlas we found 16 probes that had previously been associated with BP, 142 for SBP, 191 for DBP, and 151 probes for hypertension. Intergenic CpGs cg18264657 and cg11710912 annotated to the *ARGLU1* gene that were previously associated with hypertension in other study populations of European and South Asian ancestry were associated with renin concentration and ARR in our Ghanaian study population (Table 4). Intergenic cg05376465, previously associated with SBP in European and South Asian ancestry population, was associated with aldosterone concentration in our study. 65 CpGs previously reported to be associated with eGFR in populations of European and African American ancestry were assessed in our Ghanaian population, of which three were associated with renin concentration and one with ARR. Intergenic CpG cg01695994, cg02059849 located in the body of *PTP4A3* gene and cg22623033 annotated to the *PIP5K1C* gene were significantly associated with renin concentration, as was cg02059849 (*PTP4A3*) to ARR.

**Table 4:** Replicated differentially methylated positions associated with plasma renin, aldosterone concentration and aldosterone/renin ratio, based on candidate-gene approach

	Number of CpGs replicated*	CpG	chr	pos	Gene symbol	Gene group	Regression.Coeff.**	FDR adj. *p*-value
*Renin*								
Hypertension	1/148	cg18264657	chr18	4,455,862	intergenic		− 0.1812	0.0169
eGFR	3/65	cg01695994	chr17	80,246,403	intergenic		0.1498	0.0012
		cg02059849	chr8	142,437,898	*PTP4A3*	Body	0.0937	0.0102
		cg22623033	chr19	3,653,592	*PIP5K1C*	Body	0.0736	0.0142
*Aldosterone*								
SBP	1/136	cg05376465	chr14	76,734,327	intergenic		− 0.4734	0.0496
*ARR*								
HTN	1/148	cg11710912	chr13	107,217,605	*ARGLU1*	Body	0.1496	0.0389
eGFR	1/65	cg02059849	chr8	142,437,898	*PTP4A3*	Body	− 0.1016	0.0073
RAAS	1/45	cg12841684	chr4	149,251,768	*NR3C2*	Body	− 0.1276	0.0113

^*^Number of CpGs replicated of total number of CpGs included in candidate-gene approach

^**^For *M* values, adjusted for covariates

*eGFR* Estimated glomerular filtration rate, *ARR* aldosterone-to-renin-ratio, *SBP* Systolic blood pressure, *HTN* Hypertension, *RAAS* Renin–angiotensin–aldosterone system

Next, we performed association analysis on a subset of 45 probes annotated to *ACE*, *REN, CYP11B2, HSD11B2,* and *NR3C2* genes*,* which are all involved in the RAAS system. One of these CpGs, cg12841684 located in the body of the *NR3C2* gene, was associated with ARR at an FDR adjusted *p*-value of 0.01.

### Biological relevance

Generally, methylation levels as reported in the iMethyl database were similar compared to average methylation betas in our population (Additional file 1: Table S1). Additionally, for three CpGs associated with aldosterone, higher levels of methylation in the body of the *SKIL* and *PTPRN2* gene were associated with higher gene expression, whereas higher levels of methylation in the body of the *MLYCD* gene were associated with lower gene expression.

In the pathway analysis using the KEGG catalogue, the top 20 pathways associated with renin, aldosterone, and ARR were involved in fatty acid metabolism, diabetes cardiomyopathy, and immunological processes (Additional file 1: Table S2). However, none of the pathways were statically significant at an FDR-adjusted *p*-value < 0.05.

None of the identified significant DMPs showed a gap in Beta value distribution in the *gaphunter* analysis, indicating no signs of genetic variation influencing methylation levels of these DMPs.

## Discussion

### Key findings

In this first EWAS on plasma aldosterone and renin concentrations, we identified one DMP associated with renin concentration, ten DMPs associated with aldosterone concentration, and one DMP associated with ARR in a Ghanaian population. These DMPs contributed to 15%, 46%, and 19% of the explained variance in renin, aldosterone, and ARR concentration, respectively. Additionally, DMPs associated with renin, aldosterone, and ARR explained a substantial percentage of the variance in SBP and DBP. For the genome-wide significant DMPs associated with renin and aldosterone concentration, no differences in methylation levels were observed between participants residing in rural and urban Ghana and Ghanaian migrants in Europe. Two DMRs associated with renin and one DMR associated with aldosterone concentration were identified. We found CpGs previously associated with hypertension and kidney function to be associated with concentrations of renin and aldosterone, and we found one DMP annotated to the *NR3C2* gene of the RAAS system to be associated with ARR.

### Discussion of the key findings

We found several DMPs associated with concentrations of aldosterone annotated to genes that have previously been reported to be related to CVD risk factors. For instance, hypermethylation in the *PTPRN2* gene has been associated with metabolic syndrome in African American population [23], and hypermethylation of the *SKIL* gene has been associated with atherosclerosis [24]. We identified one DMP, cg21178689, annotated to the *KCNT1* gene, which codes for potassium sodium-activated channel subfamily T, that, among others, regulates insulin secretion, heart rate, and smooth muscle contraction [25]. Methylation in this gene has previously been associated with BMI [26] and type 2 diabetes mellitus [27]. For renin, the significant DMP was located in an intergenic region; however, the nearby located gene *PTDSS2*, has previously been shown to relate to fat mass if expressed in adipose tissue [28]. DNA methylation of cg15602420, located downstream of the *OTUD3* gene, was associated with ARR. Methylation in the *OTUD3* gene has previously been associated with glucocorticoid exposure [29]. Additionally, the significant DMRs were annotated to genes associated with BMI [30], diabetes mellitus and coronary heart disease, and the pathway analysis suggested enrichment of pathways involved in CVD pathophysiology. These findings link hormones of the RAAS to other metabolic systems.

As this is the first EWAS on the RAAS, we could not replicate our findings nor could we replicate findings from previous studies. However, in our candidate approach, we found that several DMPs previously associated with hypertension and kidney function were also associated with concentrations of renin, aldosterone, and ARR. This suggests the potential shared regulatory mechanism. This is also supported by our findings that the top DMPs associated with plasma renin and aldosterone concentration explained a substantial percentage of variance in SBP and DBP. Remarkably, however, was that we were able to replicate only one CpG annotated to genes involved in the RAAS, namely cg12841684 annotated to the *NR3C2* gene, which encodes for the mineralocorticoid receptor mediating the actions of aldosterone on salt and water balance [31]. Previous findings from animal studies have shown the impact of salt intake, hypertension, and proinflammatory cytokines on methylation and expression of the *AGT* and *CYP11B2* gene [32]. It could therefore be that the association between DNA methylation of RAAS genes and the aldosterone and renin concentration are masked in our study, because of the complex regulation of these concentrations by other factors like salt intake, serum potassium concentrations, and sympathetic nervous system activity, or because of the small sample size of this study (i.e. false negative findings).

Hypertension is more prevalent among SSA migrants in Europe compared to their non-migrating counterparts in SSA [2], and DNA methylation has been shown to differ between migrant and non-migrant populations [33]. However, the mean methylation levels for our DMPs associated with aldosterone, renin, and ARR did not differ between participants residing in rural and urban Ghana, and Ghanaian migrants in Amsterdam (Additional file 1: Table S3). This is noteworthy, as the median levels of renin seem to be lower in Amsterdam than in urban Ghana and rural Ghana, whereas aldosterone showed an opposite trend, being highest in Amsterdam and lowest in rural Ghana. This may suggest that other factors like BMI or BP impact renin and aldosterone more directly than DNA methylation does.

Hypertension in SSA origin populations is often characterised by suppressed renin and high ARR [29]. Fitting the EWAS for suppressed versus unsuppressed renin, and for high versus low ARR, classified based on median split of both distributions, showed the same DMPs that were epigenome-wide significant in the EWAS for continuous traits (data available on request). This supports the robustness of our findings and indicates that the association between renin concentration and ARR and DNA methylation is linear.

### Strengths and limitations

This is the first EWAS on hormones of the RAAS and it was conducted in a genetically homogenous populations of Ghanaians, a SSA population with a high prevalence of hypertension. Data collection was highly standardised, allowing for high-quality data and comparison of populations residing in different locations.

Our study has some limitations. Firstly, as this was an explorative study, the sample size and study design were not based on specific statistical power to detect certain effect. However, the goal of this proof-of-principle study was to lay a foundation, which can lead to further research. Secondly, we used whole blood samples to assess DNA methylation associated with aldosterone and renin concentration. DNA methylation is tissue-specific, and even though renin and aldosterone are hormones that are secreted into the blood, it is possible that DNA methylation is different in the tissues where these hormones are produced and/or act, i.e. in the kidney and adrenal glands. The DNA methylation levels for our top DMPs, however, generally showed concordance between blood, kidney, and adrenal gland tissue [34]. Thirdly, it has been shown that renin’s precursor prorenin can be activated if samples are not frozen quickly after collection, or thawed slowly before analysis [35, 36]. This cryoactivation of prorenin could result in increased renin concentration. However, as samples were frozen quickly after collection, and were thawed according to standard operating procedures, the impact of activating prorenin is likely to be minimal. Fourthly, since our study lacks genetic profiles, we were not able to exclude participants from our study with an underlying genetic defect known to be associated with changes in RAAS, such as Liddle syndrome or familial hyperaldosteronism (37). However, as these syndromes are rare, we do not expect these to have substantially impacted our findings. Additionally, results from the  *gaphunter* analysis did not indicate potential genetic variations underlying our significant DMPs. Lastly, as this study used cross-sectional data, inference on causality should be made with caution.

### Perspectives

The findings of this proof-of-principle study suggest a role of DNA methylation in the regulation of plasma renin and aldosterone concentration, with a potential link to blood pressure and other CVD risk factors. This study can serve as a starting point to further elucidate the regulation of RAAS and the pathophysiology of (salt-sensitive) hypertension, and the complex gene-environment interaction affecting these mechanisms. Specifically, future research should include larger sample size, of different ethnic origins, and preferably of longitudinal study design, in order to replicate our findings, determine ethnic-specific differences, and establish causality in relation to incidence of hypertension and CVD risk. Ultimately, this information could inform targeted interventions aiming to reduce the burden of hypertension in general, and among SSA populations specifically.

## Conclusions

In this first EWAS on renin and aldosterone concentrations in general, and specifically in a SSA population, we detected several epigenome-wide significant DMPs. Our results need to be replicated in large cohorts of different ethnic origins. Additionally, translational and longitudinal studies are needed to better understand the role of DNA methylation in the regulation of hormones of RAAS, thereby disentangling the pathophysiology of salt-sensitive hypertension in SSA populations.

## Supplementary Information

**Additional file 1:** **Table S1** Relationship between DNA methylation of differentially methylated positions and gene expression as reported in the iMEHTYL database. **Table S2** Enrichment analysis for the top 20 pathways associated with renin, aldosterone and ARR. **Table S3** Median methylation beta levels of differentially methylated positions, stratified by geographical location of residence. **Fig. S1.** Participants inclusion flow chart. **Fig. S2** QQ plots of bacon adjusted *p*-values for renin, aldosterone and ARR population. **Fig. S3** Differentially methylated region (DMR) annotated to chromosome 6 (*NFYA*) associated with renin concentration. **Fig. S4** Differentially methylated region (DMR) annotated to chromosome 6 (*RNF39*) associated with renin concentration. **Fig. S5** Differentially methylated region (DMR) annotated to chromosome 6 (*HLA-DRB1*) associated with aldosterone concentration.

## Data Availability

The datasets used and/or analysed during the current study are available from the corresponding author on reasonable request.
